# Label-Free Target Discovery Strategy for Natural Active Products

**DOI:** 10.3390/biom16040507

**Published:** 2026-03-27

**Authors:** Lei Shan, Yujia Chen, Xiuling Cao, Xuejiao Jin, Beidong Liu

**Affiliations:** 1 State Key Laboratory for Development and Utilization of Forest Food Resources, Zhejiang A&F University, Hangzhou 311300, China; c585775sl@126.com (L.S.); hcyj1849015698@163.com (Y.C.); cxiuling@cau.edu.cn (X.C.); 2 Zhejiang Key Laboratory of Non-Wood Forest Products Quality Regulation and Processing Utilization, Zhejiang A&F University, Hangzhou 311300, China

**Keywords:** non-probe-based methods, target identification, natural compounds, lysates, high-throughput, proteomics

## Abstract

The growing interest in harnessing natural compounds for health and medical applications underscores the necessity for innovative approaches to decipher their mechanisms of action. Among diverse strategies, non-probe-based methods for identifying the molecular targets of compounds represent one of the frontiers in drug discovery. This review focuses on an array of non-probe techniques for unveiling interactions between natural molecules and biological targets. The advantages and limitations of label-free protein target identification schemes suitable for lysed cells and living cells are analyzed. High-throughput target screening methods and their role in facilitating a holistic understanding of compound–target interactions are summarized. Based on comprehensive evaluation and comparison, this review aims to provide guidelines for selecting appropriate non-probe strategies to accelerate the characterization of the therapeutic potential of natural compounds.

## 1. Introduction

Natural products are a class of bioactive compounds derived from animals, plants, or microorganisms [1]. Compared with synthetic compounds, natural products have more diverse skeleton types, richer functional groups, and more complex stereo configurations, which endow them with unique biological properties [2]. Over the past 40 years, natural products have become an important source of new drugs for various diseases [3,4,5,6]. Interactions with intracellular protein targets are fundamental for natural compounds to exert their pharmacological activity. Therefore, target identification is a critical step in the discovery and development of new natural drugs [7,8]. Target protein identification methods currently include two categories: chemical probe and non-probe approaches [9]. In the former, natural product molecules are chemically modified by attaching reporter tags (e.g., biotin, and fluorophores) or reactive groups. Among these, biotin is extensively utilized due to its high binding affinity for streptavidin [9]. Cells are treated with the labeled compound to allow the molecule to bind to its target proteins and then lysed to isolate its interacting targets. Finally, a proteomics assay enables the identification of the target proteins. The foremost challenge of chemical probe methods is preserving the bioactivity of the natural product, which severely constrains feasible modification strategies. This limitation primarily stems from the requirement that any introduced modification must avoid excessive steric hindrance at the attachment site to avoid disrupting the compound’s interaction with its target protein. Moreover, certain modifications may alter the conformation, membrane permeability, or subcellular distribution of a natural product. These changes can result in the failure to identify true physiological targets or in the identification of non-specific protein interactions [9,10].

Alternatively, considering that most proteins fold into their native conformation through non-covalent interactions, and that the interaction of small-molecule compounds with their target protein can change the latter’s structure and stability, the main principle of target identification in non-probe methods is to detect changes in protein stability before and after the addition of ligand compounds [10]. Non-probe approaches do not introduce additional covalent modifications, reflecting interactions in the natural state. This also prevents false-negative results caused by probe fluorescence quenching, which can lead to a decrease in signal and the misclassification of targets as unbound targets. To address the problem of high background noise and high probe non-specific binding due to high protein concentration after cell lysis, non-probe methods utilize the physical properties of proteins in combination with quantitative mass spectrometry (MS) analysis, which can effectively distinguish between specific and non-specific binding background signals and can greatly reduce the risk of false positives. The use of non-probe methods to find targets in living cells does not require complex modifications to the cells, preserves the integrity of the cellular physiological environment, and allows for a better understanding of dynamic interactions—such as those dependent on post-translational modifications or confined to specific subcellular compartments; without the need to introduce probes that may be toxic, non-probe approaches are less toxic to cells and more suitable for prolonged observation and experimentation [11]. The combination of high-throughput target screening and non-probe methods allows for the simultaneous identification of a large number of compounds across the proteome in a short period of time. Moreover, non-probe methods typically do not require substantial time for probe labeling and optimization, making the experimental process more efficient. Furthermore, they reduce the consumption of large amounts of probe-labeling reagents, lowering experimental costs. The reduction in step complexity also decreases the likelihood of human error, enhancing the consistency of experimental conditions [12,13]. Therefore, non-probe methods are considered better for target identification. According to the underlying principle, the analysis of change in thermal stability and protein hydrolysis sensitive, chemical denaturant-and acidic reagent-induced stability, and other protein structures and properties after the addition of ligand compounds ultimately enables target protein identification.

Various classical probe-modification-free protein target identification methods have been developed. Techniques such as cellular context thermal shift assays (CETSAs) exploit the principle that drug binding stabilizes target proteins, rendering them less susceptible to thermal denaturation [14]. In addition, methods such as thermal proteome profiling (TPP) have also been developed based on changes in protein thermal stability [15,16,17]. Thermal proteomic methods have become core strategies to identify targets of natural products, successfully applied to quercetin, EGCG, celastrol, gambogic acid, and many other botanical compounds [18,19,20,21]. The newly identified targets via label-free CETSA/TPP are highly specific and closely associated with their pharmacological activities. For instance, oridonin, a diterpenoid isolated from *Rabdosia rubescens*, was confirmed to bind nucleolin—a target unreported by prior methods. This binding directly inhibits ribosome biogenesis and induces tumor cell apoptosis, clarifying the anti-cancer mechanism of oridonin [18]. Gambogic acid was found to target canopy FGF signaling regulator 3 (CNPY3), a non-druggable protein that regulates pyroptosis in prostate cancer cells, unraveling the unique anti-tumor pathway of this natural product [15]. Furthermore, ligand binding often causes conformational changes in proteins, altering their susceptibility to proteolytic cleavage. This principle underpins several techniques, including the pulse proteolysis (PP) method [22,23,24], limited proteolysis (LiP) [25], and the drug affinity responsive target stability (DARTS) method [26]. They have been successfully used in many traditional Chinese medicine monomers such as curcumin, oridonin, sanguinarine, quercetin, and tanshinone to address the challenges posed by the unclear mechanisms and targets of natural products [27,28,29,30]. For instance, curcumin can block the NF-κB signaling pathway by binding to the DNA-binding domain of the NF-κB p65 subunit, thereby explaining its anti-inflammatory and anti-tumor molecular mechanisms [27]. Quercetin inhibits the activity of heat shock protein 90 (HSP90) by binding to its N-terminal ATP-binding domain, thereby exerting anti-tumor and anti-inflammatory effects [28]. Proteomic methods, such as stability of proteins from rates of oxidation (SPROX) and solvent-induced protein precipitation (SIP), offer additional routes for target identification by measuring ligand-induced changes in susceptibility to chemical denaturation or organic solvents, respectively. SPROX verified that staurosporine can specifically bind to the ATP-binding pocket of kinases in the serine/threonine kinase family, such as CDKs, PKA, and GSK3β, inhibit kinase phosphorylation activity, block cell cycle progression and proliferation signaling pathways, and exert anti-tumor activity [31]. SIP, for the first time, verified that geldanamycin (GA), a benzoquinone ansamycin natural product, can bind to NADH dehydrogenase flavin protein 1 (NDUFV1), directly inhibit the catalytic activity of mitochondrial complex I, and ultimately cause mitochondrial oxidative stress and damage [32]. They have also been successfully applied to target identification for other natural products, providing an important tool for the analysis of the mechanism of action of natural products and the development of new drugs [10,31,33]. The newly identified target proteins of natural compounds by the above-mentioned classical methods are summarized in Table 1.

These probe-free target identification methods can be applied to natural, unmodified small molecules. In addition, large quantities of purified proteins are not required, and protein samples can be obtained from cell lines or tissue specimens. They can be used not only to validate known targets of compounds but also to discover new targets from the complex composition of natural and synthetic drugs. These approaches not only have been widely applied for drug target identification but also have been constantly improved and optimized. However, there is still room for development in research on classical probe-modification-free protein target identification, and many new methods have emerged. This review focuses on no-modification target identification methods that have been developed in recent years, classified into three groups according to their application: target identification in living cells, target identification in cell lysates, and high-throughput target identification. The latest advances and applications are summarized, and the strengths and limitations of each method are also discussed. This review aims to provide information on selecting appropriate strategies for identifying potential human disease-related target proteins of natural compounds.

## 2. Methods Suitable for Target Identification in Cell Lysates

Cell lysis is a common method for finding target proteins of small-molecule compounds, thus providing an important theoretical basis for drug discovery, disease treatment, and other fields [31,34]. It not only facilitates the release of various proteins and other molecules from the cell, making target proteins easier to detect and analyze, but also removes cell membranes and other cellular barriers that may block interactions between small molecules and their targets. Moreover, small molecules trigger comprehensive metabolic and signaling network responses in living cells under drug treatment [35]. Proteins identified through heat stability screening thus encompass both direct targets and indirect effector proteins whose stability shifts due to altered post-translational modifications or protein interactions downstream [36,37]. In contrast, in lysate-based assays, the absence of intact cellular metabolism and signaling pathways allows drugs to act directly on protein mixtures. Consequently, proteins identified through stability changes in these assays are more likely to represent direct drug-binding candidate targets. Some target identification methods require high protein concentrations, which can only be achieved by lysing cells and concentrating their contents. Thanks to efforts to address these limitations, the methods for identifying target proteins after lysing cells have been widely applied and improved over the years.

### 2.1. Matrix Thermal Shift Assay (mTSA)

Alterations in the thermal stability of proteins are often used to study ligand binding, such as the early CETSA, which is a method based on recognizing ligand-induced differences in the thermal stability of target proteins [14]. With the continuous development of technology, researchers have combined the CETSA with multiplexed quantitative mass spectrometry [38] to overcome the limitations of this assay in terms of throughput and sensitivity. The result is thermal proteome profiling (TPP), a method mostly used to find unbiased target proteins inside and outside of cells [39]. It has been applied to detect changes in protein stability in tissue samples from the organs of drug-delivering animals, thus providing comprehensive and effective proteomic information [40]. In addition, it overcomes the limitations of the traditional CETSA, such as antibody dependency in detecting target proteins and lower quantitative accuracy due to signal intensity variations [41,42]. However, the experimental design of TPP typically involves the parallel processing of dozens of samples, which are then fractionated through various steps, requiring four to six days of data collection for the mass spectrometer analysis session alone. These samples are labeled with tandem mass-tagging (TMT) reagents, leading to a dramatic increase in experimental costs when TMT reagents are used in large-scale experiments [43].

**Figure 1:**
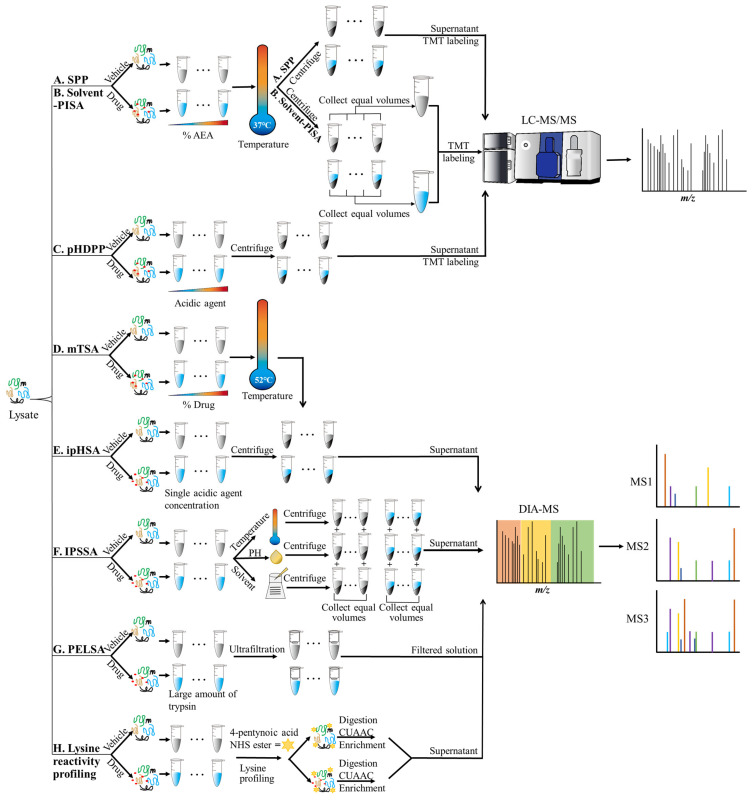
Label-free target identification methods for use following cell lysis. (**A**) SPP: Cell lysates are divided into multiple aliquots and incubated separately with the vehicle and drug. The samples are treated at 37 °C by increasing the concentration of the added organic solvent, AEA, and equal volumes of each soluble fraction are collected for mass spectrometry analysis. (**B**) Solvent-PISA: It is based on the SPP principle, where many AEA-treated samples with increasing concentration gradients are pooled for mass spectrometry. (**C**) pHDPP: Cell lysates are incubated with the vehicle or drug; then, then the concentration of acidifier is gradually increased to lower the pH, and the soluble fraction is selected for mass spectrometry after protein precipitation; (**D**) mTSA: Cell lysates are incubated with the vehicle or drug; then, proteins are denatured and precipitated at 52 °C, and finally, the soluble fraction is selected for mass spectrometry analysis. (**E**) ipHSA: Cell lysates are incubated with the vehicle or drug; then, proteins are precipitated with a single concentration of an acidic preparation, and finally, the protein abundance in the collected supernatants is measured using the DIA-MS method; (**F**) IPSSA: Cell lysates are incubated with the vehicle or drug; the soluble supernatant fractions of cell lysates are pooled after denaturation at a single denaturation condition by using elevated temperature, an acidifier, or organic solvent; protein abundance is determined with DIA-MS. (**G**) PELSA: Cell lysates are incubated with the vehicle or drug, followed by the hydrolysis of proteins with large amounts of trypsin, and finally, peptide abundance is quantified with DIA-MS. (**H**) Lysine reaction profiling: Cell lysates are incubated with the vehicle or drug, followed by the labeling of the primary amines of lysine residues in both sets of samples; then, digestion, CuAAC reaction, and enrichment are performed prior to DIA-MS analysis.

Recently, an improved method based on thermal shift, called the matrix thermal shift assay (mTSA), has been used for the efficient deconvolution of drug-binding targets and the calculation of binding affinity [44]. In the mTSA, cell lysates are treated with various concentrations of compounds, and for each condition, many technical replicates are performed vertically. The cell lysates are then denatured at a constant temperature of 52 °C to induce protein precipitation. Subsequently, the soluble fractions were selected and analyzed with high-resolution mass spectrometry in data-independent acquisition (DIA) mode (Figure 1D, Table 2) [45]. TMT and DIA are both relative quantitative proteomics analysis techniques that can be used to compare the relative differences in protein content between different samples [46]. TMT uses the isotope-specific labeling of peptide amino groups, and by detecting the intensity of the mass spectrometry tags, it can simultaneously compare the relative protein content in different samples [47]. In contrast, DIA does not rely on tags and collects secondary ion fragment information for protein identification and quantification in a data-independent scanning mode, eliminating the need for fractionation steps and any complex labeling [48]. TMT cannot distinguish the presence or absence of proteins between different groups and is inferior to DIA in the identification of low-abundance proteins [49]. Moreover, the TMT technique may lead to a certain degree of randomness in the collection of mass spectrometry data, which can result in unstable identification outcomes [49]. The DIA method, however, can obtain all fragment information of all ions in the sample without omission and without bias [50], a key issue affecting the quantitative accuracy and sensitivity of TMT-based quantification. In summary, DIA is a more direct and efficient method than the TMT technique. These improvements enhance screening sensitivity and speed up the entire target protein search process by eliminating the fractionation and labeling steps. The method has been evaluated using staurosporine, a pan-kinase inhibitor, as a benchmark. Staurosporine is a microbial-derived natural product that exhibits a wide range of biological activities and is widely employed as a chemical tool or positive control for the development and validation of novel target screening platforms [39,51,52,53]. Using this technique, the researchers identified the binding proteins of three organic acid metabolites (succinate, fumarate, and lactate) in HeLa cell lysates and also identified two previously unreported lactate-binding proteins [54]. Apart from natural products, the environmental persistent pollutant perfluorooctanesulfonic acid (PFOS) [55] has also been successfully targeted with the mTSA. In addition to validating several known targets, this assay identifies a number of potential targets for PFOS through which it potentially exerts its toxicity through these targets [44].

In short, the mTSA has greater screening sensitivity than traditional TPP, as well as substantially lower running and time costs, due to the absence of the complex fractionation steps of the latter procedure. More importantly, the mTSA can also quantify the target-binding affinity of ligands, which offers the possibility of finding the major binding proteins for ligand function. It is a suitable method, especially for studying the rapid deconvolution of ligand-bound targets as well as quantifying the binding affinity [44].

### 2.2. Solvent Proteome Profiling (SPP)/Solvent Proteome Integral Solubility Alteration (Solvent-PISA)

Organic solvents can also induce protein denaturation and precipitation. The latter is achieved by lowering the dielectric constant of the system and thus weakening the stabilization of the protein surface charge, as well as using competitively binding water molecules to disrupt the hydration layer of proteins. This is different from the principle of protein precipitation caused by thermal deformation [67]. The advantages of organic solvents for target identification are as follows: On the one hand, methods based on thermal denaturation cannot be applied to target proteins that are unresponsive to temperature, such as BCR-ABL kinase, which does not respond to thermal denaturation when bound to dasatinib [39]. In such cases, the solvent-induced precipitation method can serve as an alternative. On the other hand, the thermal sensitivity of some proteins can only be captured under extreme temperature conditions. For example, the temperature threshold for the thermal stability of the binding between DCK22 and MetAP2 is higher than that of the traditional thermal denaturation method [68]. With the improvement of organic solvent-induced protein denaturation methods, combined with quantitative proteomics for analysis, a method called solvent proteomic profiling (SPP) has been developed. Target involvement is determined based on mass spectrometry detection by analyzing the denaturation profile of the solvent, enabling the identification of the specific target of the ligand. The denaturation curve uses a melting curve to fit the protein abundance data. Specifically, the cell lysate is divided into aliquots in the presence or absence of compounds. After incubation with increasing concentrations of the organic solvent acetic acid (AEA) at a constant temperature of 37 °C, equal volumes of each soluble fraction are collected separately and subsequently subjected to TMT labeling and LC-MS/MS analysis (Figure 1A, Table 2) [56]. At the level of proteomics analysis, SPP can systematically resolve the targeting effects of small bioactive compounds [56]. The known targets of the p38 MAP kinase inhibitor SCIO-469 [69,70] and the compound alisertib [71] were validated using SPP. Furthermore, the researchers have evaluated the robustness of this method in identifying known and previously unreported drug targets [72], including atovaquone [73,74], pyrimethamine [75], MMV1557817 [76], and OSM-S-106 [77], among others. Although currently published studies have primarily focused on target identification using tool compounds to establish and validate the feasibility and robustness of the SPP technique itself, we believe that this technology holds significant potential in the field of target identification for natural products.

In order to further improve the efficiency of thermal shift assays, a method known as solvent proteome integral solubility alteration (Solvent-PISA) has been developed [56]. In this method, many samples are incubated with different concentrations of AEA, and unlike in SPP, they are then pooled together in equal volumes. The area of the sample mixed under the solvent melting curve is almost equal to the area of all the quantified proteins, so only a single TMT reagent label is required (Figure 1B, Table 2). Based on this technical approach, the degree of involvement of target proteins can be effectively assessed by analyzing the difference in protein levels between the treated and vehicle groups. In brief, the binding of target proteins can be determined by analyzing the protein levels between the compound and the vehicle-treated samples and then calculating using the *t*-test. The known targets of SCIO-469 [70] and the discovery of additional potential targets [56] have been validated using Solvent-PISA.

Overall, SPP is a suitable target identification method for small molecules that do not respond to thermal denaturation upon binding to target proteins. Solvent-PISA, as an improved version of the SPP, has the advantage of being able to cope with more challenging experimental setups, handling many replicates and different concentration gradients simultaneously. This improvement significantly increases throughput and optimizes experimental efficiency while maintaining data reliability.

### 2.3. pH-Dependent Protein Precipitation (pHDPP)

It is known that acidic pH can also induce the denaturation and precipitation of proteins [78]. Based on this, a denaturation method called pH-dependent protein precipitation (pHDPP) has been developed. It is used for proteome-wide compound target identification by detecting ligand-induced differences in receptor stability [57]. Moreover, it is an alternative approach suitable for proteins that are insensitive to thermal and solvent denaturation.

In this method, lysates from compound or vehicle incubations are equally divided into many samples, which are then treated with progressively higher concentrations of acidic reagents. Proteins are denatured and precipitated as a result of the progressively lower pH values and become insoluble in the cell lysate. However, target proteins bound to their ligands are more resistant to the effects of pH; thus, they are less prone to precipitation and are retained more in the soluble-state supernatant. Finally, the LC-MS/MS analysis of the supernatant is performed to determine the target proteins by quantifying the change in protein contents in the compound and vehicle groups at different acid concentrations (Figure 1C, Table 2) [57,79].

pHDPP has demonstrated excellent performance in the identification of several ligand–target proteins. For example, dihydroartemisinin (DHA), a natural product serving as the primary derivative of artemisinin, is widely clinically used for treating malaria and demonstrates significant efficacy in the treatment of various cancers [80,81,82,83,84]. A previous study utilized pHDPP to determine the target landscape of DHA, identifying a total of 45 potential target proteins [57]. Among the DHA candidate targets predicted through extensive molecular docking simulations, pHDPP successfully validated the ALDH7A1 and HMGB1 proteins, and a CETSA further confirmed that these two proteins indeed bind to DHA [57]. Beyond natural products, pHDPP has been used to validate the specific binding of synthetic compounds, including methotrexate, to dihydrofolate reductase (DHFR) [85] and the kinase inhibitor SNS-032 to its target CDK2 [86].

In conclusion, pHDPP is an effective and stable method for determining the mechanism of drug action and validating potential compound targets. It is an alternative approach for discovering protein targets, addresses the issue that some proteins are insensitive to heat denaturation and solvent denaturation, and also has a wide range of applications in the field of drug discovery and mechanism analysis.

### 2.4. Iso-pH Shift Assay/Integrated Protein Solubility Shift Assay (ipHSA/IPSSA)

There are also methods based on altering the pH value to search for protein targets [57]. The above-mentioned methods based on heat-induced and pH-induced protein denaturation require TMT labeling, which increases sample consumption, mass spectrometry time, and material cost. Therefore, an improved method that does not utilize TMT labeling, called the Iso-pH Shift Assay (ipHSA), has been developed. In this method, only one concentration of acidic reagent is used to lower the pH and induce protein precipitation; the change in protein solubility following ligand or vehicle addition is analyzed, and then, proteins are quantified by using DIA (Figure 1E, Table 2) [51]. Cell lysates from compound or vehicle incubations are divided into many samples in equal volumes, which are then treated with a fixed single concentration of an acidic reagent; proteins are denatured and precipitated due to the decreasing pH. Subsequently, proteomic analysis is performed using data-independent acquisition mass spectrometry (DIA-MS), and ligand-target interactions are determined by comparing changes in the abundance of each protein in samples treated under different conditions. The use of DIA effectively suppresses the generation of quantitative bias, unlike the TMT quantification technique, where a ratio compression effect is caused by the limitation on the number of isotopically labeled channels [50].

In addition to ipHSA, an integrated protein solubility shift assay (IPSSA) method, which integrates the principles of the isothermal shift assay (iTSA) [59], ipHSA and isosolvent shift assay (iSSA) [51] has been developed. First, as with the ipHSA, the ligand or vehicle is incubated with the protein sample. The sample is divided into several aliquots, each subjected to one of three distinct denaturation conditions: thermal, acidic reagent-induced, and organic solvent-induced denaturation. Afterward, soluble proteins are separately isolated under each condition, and equal volumes of the supernatants from the three conditions are then combined. Finally, the significant differences in protein abundance between the sample ligand and carrier groups are calculated by mass spectrometry analysis (Figure 1F, Table 2). In target identification for the natural product staurosporine, the IPSSA outperformed each of the three methods in the number of positive kinase targets recognized. The simultaneous use of three protein denaturation methods breaks through the detection limitations of a single method and achieves a systematic increase in proteome coverage [87]. In addition, by increasing the number of IPSSA replicates and comparing the results with those of staurosporine target proteins detected using TPP, it was found that the number of overlapping target proteins detected by the two methods increased significantly as the number of IPSSA replicates increased. Thus, by increasing the number of repetitions, it is possible to capture target proteins that are difficult to detect because of the weak solubility changes caused by ligand binding.

Overall, the ipHSA target identification method, which does not utilize TMT markers, has the advantage of being more efficient and easier to operate than the other methods, while the IPSSA shows higher sensitivity in target screening than the individual ipHSA, iTSA, and iSSA methods. In addition, the IPSSA can be implemented with a simplified experimental design by merely increasing the number of sample replicates to increase the positive rate of target screening. It also improves throughput and reduces costs compared with the other methods, making it widely applicable as a fast and effective tool for drug target identification.

### 2.5. Peptide-Centric Local Stability Assay (PELSA)

Alterations in protein hydrolysis sensitivity are often used to study ligand binding, and they are based on the fact that ligand binding to the target protein alters the thermodynamic stability of the latter and affects the protein hydrolysis sensitivity upon protease incorporation [26,88,89]. In such a method, post-hydrolysis proteins are digested into numerous peptides. The hydrolyzed peptides are predominantly analyzed via Western blotting to determine target protein abundance, which heavily relies on antibody reliability and is prone to masking subtle alterations in low-abundance proteins. Additionally, the method depends on detecting structurally complex specific peptides within the peptide library. These factors collectively lead to a significant reduction in sensitivity for target protein identification. Therefore, the peptide-centric local stability assay (PELSA), a novel, highly sensitive method, has been developed to leverage differences in proteolytic stability induced by ligand binding. It enables the precise determination of ligand–receptor’s binding regions and the quantification of binding affinity at the peptide level [58].

Experimentally, cell lysates are evenly divided into eight aliquots: four replicates are incubated with the vehicle, while the other four are incubated with the ligand. Subsequently, a high concentration of trypsin is added to directly hydrolyze proteins into small peptides. In conventional MS workflows, trypsin is used at a low concentration to achieve gradual, complete digestion into peptides suitable for identification. In the PELSA, the high-concentration trypsin creates a destructive proteolytic environment. When a critical cleavage site within the binding pocket is protected, the entire domain resists degradation. Conversely, unprotected proteins or domains are rapidly and extensively digested. Therefore, it is capable of amplifying minor local stability differences induced by ligand binding into significant peptide abundance variations through a cascade amplification effect. Meanwhile, specific proteolysis ensures that cleavage events occur at predictable sites, allowing peptides with altered abundance to be precisely mapped back to the protein sequence, thereby locating the ligand’s binding region. After digestion, the samples undergo ultrafiltration to separate the resulting small peptides from larger fragments and intact proteases, and are then collected for MS analysis with DIA quantification to determine peptide abundance differences between the ligand-treated and vehicle groups. Peptides with significantly higher abundance in the ligand-treated group are identified as fragments originating from protein regions stabilized by the ligand (Figure 1G, Table 2) [58]. Therefore, unlike conventional proteomics, which focuses on changes in global protein abundance or modifications, PELSA shifts the focus to precise changes at the peptide level.

Limited proteolysis–mass spectrometry (LiP-MS) is a mature limited proteolysis-based method that enables the simultaneous identification of target proteins and binding sites [90,91]. LiP–MS employs a ‘two-step differential proteolysis’ strategy, which involves initial limited proteolysis, followed by a second complete digestion step to ultimately generate peptides suitable for MS analysis [90]. In the first step, limited proteolysis is performed under native conditions using a non-specific protease. The proteins remain folded, making their flexible regions accessible to cleavage, while rigid domains or ligand-binding regions are protected due to steric hindrance. When ligand binding induces local conformational changes, the accessibility of cleavage sites in that region is altered (either exposed or shielded), generating a population of intermediate peptides distinct from the control group. In the second step, a strong denaturant is added to completely disrupt all higher-order structures, followed by complete digestion of the intermediate peptides using a specific protease. This produces final peptides that also differ from those in the control group. By comparing the final peptides from both sample groups via MS, those exhibiting significant abundance changes report structural alterations in their source regions, thereby simultaneously achieving target protein identification and binding region localization [90].

The two-step digestion workflow of LiP-MS is inherently complex, as the binding-informative peptides generated during the initial limited proteolysis become intermingled with the substantial background peptides produced in the subsequent complete digestion step [91]. In contrast, the one-step deep digestion strategy employed in the PELSA generates uniform peptides with low complexity. This simplicity confers superior sample tolerance and reproducibility on the PELSA when handling complex biological samples, far exceeding that of LiP-MS [58]. Moreover, LiP-MS effectively detects only those ligands that induce conformational changes resulting in the occlusion of protease cleavage sites on the protein surface. The PELSA, however, capitalizes on the ubiquitous local stability changes that accompany ligand binding, rendering it a more universal and robust approach [58]. Furthermore, the PELSA demonstrates significantly superior sensitivity compared with LiP–MS. When identifying kinase targets of staurosporine, even in comparison with the improved version of LiP–MS (LiP-Quant) [92], the sensitivity of the PELSA is enhanced approximately 12-fold [58]. Compared with LiP-MS [93], this method could identify more ligand-binding peptides for methotrexate [94] and SHP099 [95]. PELSA successfully detected the canonical protein targets of rapamycin, a prototypical microbial-derived natural product [96]. Based on this method, it was possible not only to determine that rapamycin exerts its effect by binding to FKBP1A, thereby inducing the interaction between FKBP1A and mTOR, but also to determine the binding affinity of rapamycin for FKBP1A, which was closer to literature-reported values than those measured with LiP–MS [58,97]. Furthermore, with the PELSA, researchers precisely mapped the binding region of the FKBP1A-rapamycin complex on mTOR [58]. These results collectively validate the robustness of this technology in analyzing natural product-induced protein interactions and identifying components of protein complexes.

A high-throughput analysis method based on PELSA technology, the HT-PELSA, has been developed. This strategy maintains the high sensitivity and good reproducibility of the original method while enabling batch sample processing in 96-well plates, significantly increasing the experimental throughput by up to 100-fold and effectively reducing costs [98]. Additionally, a detergent-free grinding (DFG) sample preparation method has been established to enhance the applicability of PELSA technology in identifying targets of ligand-ionotropic peptide interactions. This strategy is known as the DFG-PELSA. Compared with the traditional freeze–thaw method, DFG-PELSA improves the recovery efficiency of transmembrane proteins by approximately 2.5 times, demonstrating significant advantages in membrane protein target recognition, with performance surpassing that of conventional detergent-based strategies [99].

In summary, the PELSA is a highly sensitive, versatile, and promising limited proteolysis method. It is particularly suited for detecting subtle protein–ligand interactions by capturing stability alterations in protein regions. This capability further enhances its sensitivity in detecting weak and localized protein interactions, enabling large-scale applications in studying local binding affinity between ligands and proteins.

### 2.6. Lysine Reactivity Profiling

Modifications of the active product, such as the addition of a probe, can result in a change or loss of the functional activity of the small molecule, thus leading to failure to recognize the true target protein [10]. However, there is a class of methods that does not require modification of the natural active product, but in which the probe is also involved. Lysine reactivity profiling enables the determination of ligand–target protein binding and their binding sites at the proteomic level. Specifically, it is known that the specific binding of a ligand to a target protein alters lysine reactivity within the binding region [100]; these reactivity changes can be recognized by using an excitable lysine probe, providing the basis for the final determination of ligand–target binding [23]. In short, cell lysates are first treated with a ligand or vehicle, and then 4-pentynoic acid NHS ester is added to both mixtures for co-incubation to label primary amines with lysine residues. Here, 4-pentynoic acid NHS ester serves as a chemical probe for identifying active lysine residues in proteins. The proteins are subsequently digested into peptides, and biotin is added to the labeled peptides by using the copper(I)-catalyzed alkyne-azide cycloaddition (CuAAC) reaction [101]. The labeled peptides are enriched, and the collected supernatant is analyzed with DIA-MS. Finally, the ligand- and vehicle-treated groups are compared to test the significance of the difference in target protein abundance (Figure 1H, Table 2) [23].

The method was further used to detect the kinase targets of dasatinib that had been previously identified with TPP [39] and was found to have nearly equivalent screening sensitivity to TPP. Additionally, two previously unreported potential kinase targets were discovered, which suggests that lysine reactivity profiling has more sensitive screening properties in the search for targets of specific compounds [23]. Because this method detects changes in lysine residue reactivity, more specific information about the binding region and changes in the spatial structure of the target can be acquired than with TPP. By applying the principle of lysine reactivity, the researchers employed an optimized lysine-targeting probe combined with quantitative activity-based proteomics analysis to map the interactions between the natural products phloroglucinol meroterpenoids and lysines in breast cancer cells. From a total of 9268 quantified lysine residues, 159 lysines on 130 proteins were identified as liganded by the natural products, given interference with the function of proteins involved in lipid metabolism, mitochondrial respiration, and glycolysis [102].

In conclusion, the lysine reactivity analysis method utilizes probes while lacking the drawbacks of probe-labeled modification methods. It is based on the detection of the reactivity of lysine residues and serves as a powerful technique that complements the well-known methods, such as TPP.

## 3. Methods Suitable for Target Identification in Living Cells

Cell lysis experiments are subject to various limitations: the inability to accurately simulate the complex cellular microenvironment, the potential overlooking of protein post-translational modification, and the difficulty of fully reflecting the true function of proteins in physiological states [103]. In contrast, living cell experiments preserve the natural environment and molecular interactions within the cell, providing researchers with more direct evidence of their physiological relevance and molecular interactions. This experimental method can capture dynamic biological processes [104,105] and allow for the in-depth study of post-translational modifications of proteins [106,107], thus providing a more accurate perspective for determining the exact mechanism of action of compounds. Thus, methods suitable for target protein identification in living cells have been developed. In the following section, we summarize and discuss probe-modification-free protein target identification methods suitable for living cells developed in recent years, which are more conducive to the in-depth understanding of cellular processes and disease mechanisms and to the exploration of target and off-target effects of drugs in vivo.

### 3.1. Isothermal Shift Assay (iTSA)

**Figure 2:**
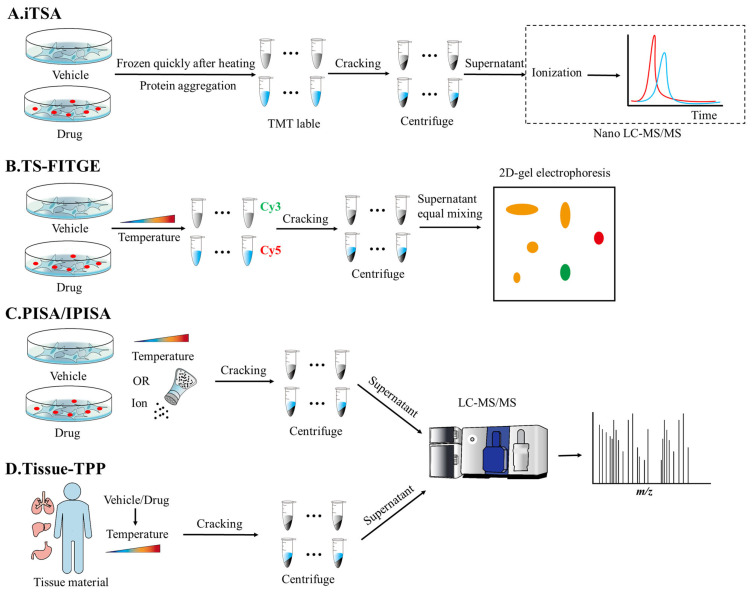
Methods suitable for target identification in living cells. (**A**) iTSA: Cells are incubated with solvents and drugs, and the samples are subjected to a single high-temperature treatment. The supernatant was collected after centrifugation for mass spectrometry analysis. (**B**) TS-FITGE: Cells are incubated with solvents and drugs, followed by temperature gradient treatment and staining with different fluorescent dyes. Then, the supernatant is collected for 2D gel electrophoresis to observe differences in the thermal stability of each protein spot; (**C**) PISA/I-PISA: After the incubation of cells with the drug, the samples are exposed to a temperature gradient or an ion concentration gradient; then, the supernatant in the cell lysate was subjected to LC-MS/MS analysis. (**D**) Tissue-TPP: Tissue samples of different types are treated with drugs and ground up. After temperature gradient treatment, the supernatant of the lysed tissue is collected for LC-MS/MS analysis. The red dots in the figure represent the ‘drug’. In 2D gel electrophoresis, the circles of different colors represent fluorescent signals from proteins labeled with different fluorescent dyes.

The iTSA method enables proteome-wide target identification in both live cells and cell lysates [59]. In this method, first, ligand- or vehicle-treated live cells or cell lysates are heated to a predetermined optimal temperature, selected to maximize the difference in protein stability between the groups. Following heating, samples are rapidly frozen to preserve the cells or cool the lysates. NP-40 and benzonase are added, and the mixture is incubated at 4 °C for 1 h. The cell membrane/organelle membrane is gently lysed to release membrane-associated proteins. After labeling with TMT, the supernatant, obtained from either cooled lysates or lysed cryopreserved cells, is analyzed with nano-liquid chromatography–tandem mass spectrometry (nanoLC-MS/MS) (Figure 2A, Table 2) [59]. Target identification is achieved by quantifying the difference in soluble protein abundance (ΔS) between treatment and control groups. Proteins exhibiting positive ΔS (ΔSm > 0) are considered stabilized by ligand binding and are thus prioritized as candidate targets. Compared with TPP, fewer samples are required because the iTSA only examines thermal stability changes at a single temperature. This reduces the overall elapsed time for the mass spectrometer. The simplification of experimental steps allows researchers to reduce data fluctuations by increasing the number of replicates, thereby making small stability changes easier to statistically identify. The combination of off-line peptide fractionation and LC-MS/MS with an ultra-high-resolution mass spectrometer [108] resulted in a significant increase in throughput [59].

Using this method, Wang T. et al. successfully constructed a comprehensive target profile for Danhong injection (DHI), identifying 110 direct targets of DHI [109]. They further delineated the specific targets of nine of its core active components (such as salvianolic acid A, protocatechuic aldehyde, and hydroxysafflor yellow A) in DHI [110,111,112]. These interactions were subsequently validated using surface plasmon resonance (SPR) to confirm specific binding and elucidate the underlying pathway [112]. In another example, Yang G. et al. identified PfTyrRS (*P. falciparum* tyryl-tRNA synthetase) as the target of the flavonoid antimalarial natural product okanin [113]. The iTSA has higher accuracy in identifying targets. Studies have shown that the ability of this essay to identify known targets that bind to staurosporine is also higher than that of TPP [59,114]. Subsequently, other researchers leveraged the simplicity of the iTSA’s single-temperature treatment, combined with the automated proteomics sample preparation platform and DIA quantification, to establish a high-throughput screening method for compound targets. The screening process can process 96 samples simultaneously in a short time, which is more than 10 times higher than the throughput of TPP. It was shown to be very accurate in target identification, identifying known targets for 20 kinase inhibitors [115]. The above study fully demonstrates the unique advantages and great potential of the iTSA in the field of natural product target identification—the assay not only enables the precise identification of direct targets within complex traditional Chinese medicine systems but also systematically elucidates the molecular basis underlying multi-component synergistic effects, thereby opening up new avenues for determining the pharmacodynamic material basis and mechanisms of action of natural products.

In summary, the iTSA can be used to search for ligand target proteins in a wide range of cell states, including live cells and cell lysates. Its simplified experimental design provides a higher-throughput alternative to other traditional target identification methods.

### 3.2. Thermal Stability Shift-Based Fluorescence Difference in Two-Dimensional Gel Electrophoresis (TS-FITGE)

Two-dimensional gel electrophoresis usually involves separating proteins by isoelectric point by isoelectric focusing and then further separating them by molecular weight with SDS-PAGE [116]. This results in a two-dimensional map of protein point distribution, with each point representing a protein. Based on this, 2D fluorescence difference gel electrophoresis (2D-DIGE) entails labeling different samples with different fluorescent dyes, mixing them together for electrophoresis, and detecting the protein spots of each sample separately using different wavelengths of excitation light. In 2D-DIGE, ‘difference’ refers to the ability to compare differences in protein expression between different samples [117]. Barbara Sitek et al. used this method to express activated TrkA and TrkB tyrosine kinases in neuroblastoma cells and identify differences in protein expression following their activation, revealing dynamic proteomic changes [118]. TS-FITGE is an innovative label-free target identification approach that integrates thermal shift analysis with fluorescence-based differential gel electrophoresis (2D-DIGE). This electrophoresis-based method can effectively quantify the changes in the abundance of a given protein in the soluble fraction [119,120]. TS-FITGE is based on the detection of changes in protein thermal stability induced by bioactive compounds, marking changes in their melting temperature (Tm). First, cells incubated with either bioactive compounds or vehicle are subjected to a temperature gradient (e.g., 42–60 °C at 2 °C intervals); then, they are quickly lysed on ice. Soluble proteins from the lysates are differentially labeled—the vehicle with Cy3 (green), compound-treated proteins with Cy5 (red), and untreated controls with Cy2 (blue). The three labeled samples are mixed in equal amounts and separated with two-dimensional electrophoresis. Protein spots showing significant differences in red/green fluorescence ratios are cut and analyzed with mass spectrometry after in-gel enzymatic hydrolysis (Figure 2B, Table 2) [60]. Thermal stability shifts can be determined based on the differences in signal intensity of the two fluorescent dyes for the same protein spot. Melting curves for each protein spot are generated across a temperature gradient, and putative target spots can be excised for mass spectrometric identification. These proteins can be further verified with CETSA to confirm their biological relevance, significantly enhancing the comprehension of protein–ligand interactions and their biological roles.

Extensive research has demonstrated the efficacy of TS-FITGE in target identification. Park H. et al. used this method to identify NPM1 as the direct target of hordenine, showing that hordenine binding increases NPM1 thermal stability and promotes translation [60]. The method’s versatility extends beyond natural product research into cancer drug discovery. Using this technique, researchers confirmed that PCYT2 is a key target whose translocation to lipid droplets induces lipophagy in SB2301-treated HepG2 cells [121]. Similarly, HSP60 was identified as the target of the anti-cancer TRIP complex with TS-FITGE [122]. In addition, the method has been employed in conjunction with other proteomic approaches to characterize the targets of natural products associated with cancer metabolism [123]. Such insights show the efficiency of TS-FITGE in identifying targets at the proteomic level and enhance our understanding of the biological activities of natural products.

Furthermore, TS-FITGE can also be used to elucidate the mechanism of cell response to various stimuli. For instance, researchers have employed this technique to determine the potential mechanism of DNA oxidative damage as a selective cytotoxic drug acting on cancer cells [124]. This information is crucial to understanding the cellular processes involved in disease pathogenesis and to the development of more targeted and effective therapies. With the increasing number of research studies employing this method, TS-FITGE is expected to play an increasingly significant role in advancing our understanding of biological processes and facilitating the development of more effective therapeutic strategies [125,126,127,128].

### 3.3. Proteome Integral Solubility Alteration (PISA)/the Ion-Based Proteome-Integrated Solubility Alteration (I-PISA)

Similar to the iTSA and TS-FITGE, proteome integral solubility alteration (PISA) is also used to identify drug targets based on changes in protein thermal stability. What makes it different is that PISA uses integration to integrate and curve-fit the protein solubility data obtained at different temperatures. The cells treated with either drugs or vehicle control are subjected to a range of temperatures (e.g., 48–60 °C). After cell lysis, the soluble supernatant is collected by using centrifugation at each temperature point, and for each treatment group, equal volumes of supernatant from all temperature points are pooled together. This pooled sample represents the integrated soluble proteome across the entire temperature range for that specific condition. Then, the abundance ratio between the drug-treated and control groups is calculated as ΔSm [log_2_ (drug/control)]. A ΔSm value greater than 0.1 is considered indicative of drug binding, marking the protein as a potential target (Figure 2C, Table 2) [62]. This method differs from Solvent-PISA in that it directly treats living cells. PISA better reflects the effects of drug treatment on cells, allowing the target of drug action to be identified. When compared with traditional techniques that rely on curve fitting of protein solubility data obtained under varying temperatures and drug concentrations, PISA uses integral thinking to analyze the area change under the entire solubility curve, rather than relying on the detection results of single-temperature-point experiments. This method can effectively eliminate the random error caused by single-point measurement and greatly reduce the false positive rate. The important application of IPSA is mainly reflected in the study of drug mechanisms. Fu Z et al. used PISA to screen for targets of alantolactone in non-small cell lung cancer (NSCLC) cells and identified AKR1C1. Alantolactone inhibits the activity of AKR1C1, blocks the synthesis of prostaglandins, and inhibits the growth of tumors [129]. Goldstein S.I. et al. used PISA to analyze the mechanism of the binding of rocaglates (natural product derivatives) to RNA helicases such as eIF4A/DDX3X, and revealed their ‘RNA-protein molecular clamp’ mode of action, providing a new strategy for the development of natural products targeting translation regulation, proliferation, and metastasis [130].

Based on PISA, I-PISA incorporates ion concentration variations. Drug-specific target proteins exhibit significantly altered resistance to ion-induced precipitation, whereas non-target proteins not bound to the drug show minimal solubility alterations under ionic influence. It also enables the comprehensive profiling of protein–molecule interactions at the proteomic level. Cells treated with drugs or the vehicle are incubated at 37 °C for 2 h and subsequently lysed on ice. The lysates are divided into aliquots and incubated with a concentration gradient of ionic solution (e.g., Na_2_SO_4_) at room temperature for 10 min to induce protein precipitation. Following centrifugation, the supernatants are collected for LC-MS/MS analysis, and ΔSm values are calculated to find proteins exhibiting differential stability upon drug binding as candidate targets (Figure 2C, Table 2) [63].

I-PISA has been listed as the core technology of label-free natural product target identification in both cell lysates and intact cell environments, which is suitable for heat-sensitive and multi-component natural products. It can be used for global target analysis of traditional Chinese medicine extracts and compounds [119]. In addition to natural products, this method has also been used in the search for targets such as anti-cancer compounds or antibiotics [131,132]. I-PISA enables whole proteome analysis, greatly increasing the chemical complexity of the sample and further realizing the depth of mass spectrometry analysis [133]. A study combining I-PISA with a machine learning framework for predicting protein interactions further exemplifies its utility in dynamic biological contexts [134]. However, the need for the interpretation of complex datasets, including specialized analytical equipment, may limit its widespread adoption in certain research settings.

### 3.4. Tissue-Thermal Proteome Profiling (Tissue-TPP)

Unlike the previously described methods, which use viable cells as sample materials, Tissue-TPP uses biological tissues as experimental materials to simulate drug metabolism in the body and can be carried out in vitro, in situ, or in vivo. It has revolutionized drug target identification and proteomics research [61]. Organs or tissues from animals were incubated with drugs; alternatively, after the treatment of individual animals, tissues are quickly taken. In either case, tissues are rapidly minced on ice and lysed by using mechanical homogenization. NP-40 is included to enhance membrane protein detection. After clarification by centrifugation, the supernatant is subjected to a temperature gradient (similar to TPP) and analyzed with MS. Soluble protein abundance is quantified at each temperature point, and the melting curve of each protein is fitted to calculate the Tm shift. Proteins with significantly increased Tm are identified as the potential binding target of the drug (Figure 2D, Table 2) [135]. This method allows for the quantification of protein abundance at the proteomic level over the entire temperature range.

Dai G. et al. used the traditional Chinese medicine bletilla striata (BSE) to treat an ovariectomized rat model and revealed that BSE exerts anti-osteoporotic effects by targeting ATM, PIK3R1, and NR3C1 [136]. Jung J. et al. confirmed that gambogic acid (GBA) can significantly induce pyroptosis in prostate cancer DU145 cells, and by using Tissue-TPP, they identified CNPY3 as its direct binding target [15]. Perrin et al. used this method to further design blood-TPP to explore the off-target effect of the Bromodomain and extraterminal (BET) family inhibitor JQ1 in treatment [135], while Wu et al. integrated Tissue-TTP with an automated proteomics sample preparation platform [115]. Beyond drug target identification, Tissue-TTP has been applied in diverse proteomic research areas and extended to the target search for other drugs, highlighting its potential in disease research [137,138,139].

Despite significant advances in the method, challenges and limitations remain. Berlin et al. found that nonionic surfactants can affect the thermal stability of proteins (especially membrane proteins), emphasizing that there may be some difficulties in using Tissue-TTP to identify membrane protein targets [140]. To circumvent the interference of non-ionic surfactants on membrane protein, alternative approaches, such as CETSA and SPR, can be employed. CETSA is performed in situ within intact cells, eliminating the need for membrane protein purification, detergent addition, and disruption of the native cellular membrane architecture. The other method, SPR, enables the immobilization of membrane proteins onto lipid-modified sensor chips to directly measure binding affinity and kinetics, thereby yielding more accurate and reliable quantitative data. However, the complexity and heterogeneity of tissue samples also represent a challenge to accurate target identification, which requires further methodological improvement and verification [141].

In summary, the iTSA, TS-TIFGE, I-PISA, and Tissue-TTP are proteomic techniques that can be used in living cells, providing greater benefits in terms of physiological correlation and dynamic information.

## 4. High-Throughput Scheme for Finding Target Compounds

Although a series of feasible target discovery schemes have been developed for lysing cells and living cells, these schemes require the separate processing of a single sample and cannot achieve the simultaneous processing of large numbers of samples. Moreover, potential manual errors can be dramatically increased, especially when handling large sample groups. To address these limitations, researchers have developed a series of high-throughput schemes to identify compound targets. These schemes enable the simultaneous detection of various compounds and targets, significantly enhancing screening efficiency. Furthermore, the experimental process can be automated, reducing manual errors and ensuring experimental reproducibility.

### 4.1. Mechanical Stress-Induced Protein Precipitation (MSIPP)

**Figure 3:**
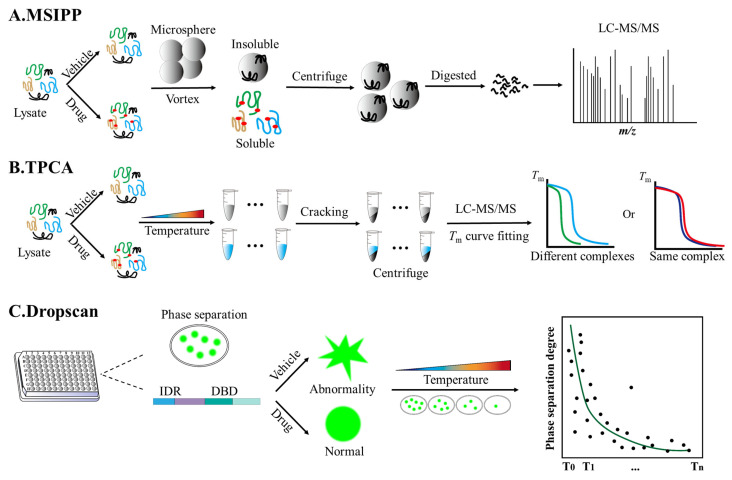
High-throughput schemes for finding target compounds. (**A**) MSIPP: Cell lysates are incubated with vehicle and drugs, and after the addition of microbeads, a mechanical stress vortex is used to promote the precipitation of non-target proteins and the combination of microbeads. The microbeads are digested and analyzed with mass spectrometry to identify the target protein of the drug. (**B**) TPCA: The samples are subjected to temperature gradient treatment. After centrifugation, the supernatant is collected for mass spectrometry analysis, and the thermal shift curves of different proteins are plotted. The proteins belonging to the same complex were determined by analyzing the thermal shift curve. (**C**) DropScan: A phase separation protein library related to cancer is constructed and treated with no loads and drug loads. Thereafter, the cells are subjected to a thermal gradient to enable subsequent high-throughput detection of drug effects on the phase separation of intracellular proteins.

As an inventive advance in proteomics and drug discovery, MSIPP represents an innovative strategy for identifying drug targets by leveraging the differential precipitation behavior of proteins under mechanical stress. The integrated workflow comprises mechanical stress-induced protein aggregation on microparticles, on-particle peptide digestion, and mass spectrometry analysis to compare insoluble protein abundance in drug-treated versus untreated samples. Experimentally, cells are collected and lysed using ultrasound. The lysates are divided into a drug group (treated with target compounds) and a control group (equal volume of vehicle), and incubated at room temperature for 40 min to allow for sufficient ligand-protein binding. Two volumes of carboxylate-modified magnetic beads are added to each protein sample, and vortexed for 80 min. During this process, the continuous mechanical force induces protein precipitation, while the magnetic beads simultaneously capture the precipitated proteins. The magnetic beads are then adsorbed using a magnetic separator and washed to remove non-specific binding proteins; they are successively re-suspended in HEPES buffer and heated to 95 °C for 5 min for complete disulfide bond reduction and thiol alkylation. Trypsin/LysC mixed enzyme is added and incubated overnight at 37 °C to digest the protein into peptides. The supernatant containing the peptide segment is used for LC-MS/MS detection (Figure 3A, Table 2) [53,64].

As previously noted, nonionic surfactants affect the thermal stability of certain proteins, especially membrane proteins, and reduce the identification of targets in the TPP [140]. Moreover, the shear stress produced by cell disruption leads to the unfolding of the protein at the air–water interface, leading to the formation of insoluble aggregates and precipitation. Notably, some detergents, such as Polysorbate 20, which have surface activity, can prevent the interactions between proteins and the air–water interface [142]. On this basis, MSIPP integrates detergent supplementation into the experimental workflow, which can help reduce the application temperature and identify the target protein more efficiently. Furthermore, this optimized condition can also improve the resolution and effectiveness of other protein target identification techniques. MSIPP can be used for target identification of small-molecule drugs, natural products, and traditional Chinese medicine extracts, especially for thermally unstable and unmodified compounds [119,143]. This modified approach had been validated for its ability to identify the known targets of several compounds (methotrexate, raltitrexed, SHP099, geldanamycin, and a protein kinase inhibitor, staurosporine), and to find a new target for raltitrexed [53].

Subsequently, the aforementioned pHDPP was developed based on MSIPP. It has been shown that MSIPP is indicative of the expanding scope of stress-induced precipitation methods, which are instrumental in facilitating a deeper comprehension of protein–drug interactions and the identification of viable therapeutic targets. The versatility and efficiency of these approaches highlight the potential of MSIPP technology to revolutionize the field of drug discovery, offering new pathways for the rapid identification and analysis of drug targets.

### 4.2. Thermal Proximity Co-Aggregation (TPCA)

TPCA is a frontier method in the field of proteomics that has revolutionized our understanding of protein complex dynamics within cells. Built upon the foundational techniques of CETSAs and TPP, this approach was first introduced by Tan et al. [65]. Its core principle relies on the phenomenon that interacting proteins undergo co-aggregation during thermal denaturation, thereby exhibiting similar solubility characteristics and melting curves. Experimentally, cells are collected and treated with a drug or a vehicle control. Then, cell suspensions are subjected to a temperature gradient ranging from 37 °C to 64 °C. Following heating for 3 min in a thermal cycler, the cells are immediately cooled on ice for 3 min to facilitate the co-aggregation of thermally unstable protein complexes. The cells are subsequently lysed, and centrifugation is performed to remove aggregated precipitates, including thermally denatured and co-aggregated protein complexes. In TPCA, quantitative mass spectrometry is used to analyze the remaining soluble proteins. This generates melting curves indicating how protein abundance varies with temperature; proteins in the same complex exhibit similar melting curves. The analysis assesses the similarity in thermal solubility profiles of protein pairs using statistical measures such as Euclidean distance or Pearson correlation, allowing for the identification of protein complexes affected by various cellular conditions—specifically including those regulated by small-molecule ligands (Figure 3B, Table 2) [144].

Based on this, Li M et al. used TPCA to find the target of veratramine (VAM), a natural alkaloid extracted from *Veratrum* plants, and successfully verified that VAM directly binds to ATP6V1C1 to inhibit V-ATPase activity. This inhibition blocked lysosomal acidification and caused autophagy–lysosomal pathway damage, ultimately leading to cancer cell death [145]. Xue Lu et al. further significantly reduced the sample input requirement for TPCA, and now, only 1 μg of protein is needed to search for target drug proteins. Using this optimized approach, the known target proteins of methotrexate and panobinostat were successfully identified, indicating the improvement in the throughput of sample processing [146].

TPCA is a high-throughput technique for monitoring the dynamic changes in intracellular protein complexes based on the thermal stability of proteins. Its core advantage is that it enables the analysis of protein interaction in the whole proteome range in non-engineered cells and tissues. Through multi-temperature thermal denaturation and mass spectrometry quantification, protein interactions and complex states can be inferred by the similarity of the melting curve. In target identification and mechanism analysis, system-level coverage of ‘from target to complex to pathway’ can be realized, which is especially suitable for in situ, high-throughput, and multi-conditional comparisons of living cells/tissues. Moreover, a known target can be used as an anchor point, and proteins covariant with its melting curve can be found by using TPCA. Subsequently, the list of complex subunits and interaction partners can be constructed. This can be used to determine whether drugs induce complex assembly/dissociation, subunit replacement, or recruitment of regulatory factors, thereby clarifying the downstream pathways and drug resistance mechanisms. This innovative approach allows researchers to map the interactions and dynamics of protein complexes in vivo, providing invaluable insights into cellular mechanisms and the molecular basis of diseases [147,148]. As the field of proteomics continues to evolve, the integration of TPCA technology with other analytical methods and computational models will likely lead to even more significant discoveries [149,150].

### 4.3. DropScan

DropScan, in addition to identifying unknown targets using label-free methods, also enables the construction of a library of disease-related proteins from known pathogenic proteins, and new drugs that can target these pathogenic proteins can be identified by screening this library with different drugs.

Chromosomal rearrangements often lead to the formation of fusion proteins, which play pivotal roles in the onset and progression of various cancers. These fusion proteins frequently contain phase separation-prone domains (PSs) and DNA-binding domains (DBDs), leading to the formation of biomolecular condensates that significantly impact gene expression and cellular behavior [66,151]. When phase separation is abnormal, non-target proteins or nucleic acids are mistakenly recruited into the condensates, resulting in an imbalance in the proportion of normal components and directly destroying the physiological function of the condensates. The originally dynamically reversible condensates lose fluidity, undergo irreversible solidification, and form abnormal aggregates. Moreover, some proteins will undergo further conformational changes to form amyloid fibril deposition. Aggregate dysfunction is associated with a variety of pathological processes. Therefore, the discovery of drugs that can interfere with such abnormal aggregates is crucial to the treatment of diseases with abnormal phase separation, such as Parkinson’s disease [152,153].

DropScan emerges as a revolutionary high-throughput screening method that helps to quickly screen drugs that regulate the formation of abnormal aggregates of fusion proteins by constructing a protein library. In a typical experimental workflow, cells stably expressing the fluorescent phase-separated protein are cultured to the desired growth stage, seeded into multi-well plates, and incubated at 37 °C for 12–24 h to allow for cell attachment and protein aggregate formation. Small-molecule compounds are added to experimental wells, with control wells receiving an equal volume of vehicle. A positive control—consisting of known protein aggregates that can be dissolved by 1,6-hexanediol—can also be included. After incubation at 37 °C for 6–24 h (adjusted according to the aggregation dynamics), aggregates are photographed using a high-content real-time imaging system. The number, area, and average fluorescence intensity of aggregates in each cell are analyzed to find a specific drug that can dissolve the aggregates (Figure 3C, Table 2) [66]. Using this technology, the drug candidate LY2835219 was shown to effectively dissolve abnormal condensates formed by Ewing‘s sarcoma fusion protein in a reporter cell line, thus highlighting the potential of targeting aberrant phase separation as a therapeutic strategy [154].

DropScan represents a shift in our approach to cancer treatment from targeting genetic and protein expression alterations to directly modulating the physical state and interactions of proteins within the cell. This shift can help to more specifically screen for drugs tailored to a specific cancer or neurodegenerative disease, thereby providing more effective and less toxic treatment options. As research continues to unravel the complexities of phase separation and its role in disease, technologies such as DropScan will be instrumental in translating these findings into tangible clinical benefits.

Many natural products, including flavonoids, alkaloids, polyphenols, and terpenoids, have been shown to regulate protein phase separation, with some being capable of dissolving pathological aggregates (TDP-43, FUS, α-syn, BRD4, etc.) [155,156,157,158,159]. However, traditional target identification methods (e.g., TPP, CETSA, PISA, MSIPP, and TPCA) cannot directly observe aggregate morphology or dynamics. DropScan, a high-content screening platform enabling real-time, quantitative, and automated analysis of condensate dynamics in living cells, overcomes this limitation and is uniquely positioned to capture the multi-target effects of natural products on aggregate-regulating pathways [66].

In summary, these methods greatly improve the efficiency of marker-free target identification and are suitable for large-scale screening and signal network research. By analyzing the interaction between proteins and the construction of protein libraries, the entire screening process is more refined, directly detects the binding of drugs to target proteins, and accelerates drug target identification and verification.

## 5. Summary

In this review, we summarize recent developments on strategies for non-probe modification target identification, including approaches that are applicable to cell lysates, techniques that are suitable for analyzing targets in living cells, and high-throughput identification methods. The advantages and disadvantages of each approach are outlined to highlight their role in providing a comprehensive understanding of compound–target interactions.

Lysing cells directly releases intracellular proteins, allowing researchers to directly analyze the entire proteome of the cell; it can also be associated with techniques such as quantifying low-abundance proteins with mass spectrometry, with high sensitivity in finding targets. The method can also be used for large-scale protein identification and quantification in combination with high-throughput proteomics techniques. However, lysed cells may not fully reflect the natural structure and function of proteins in the intracellular environment. It is difficult to capture the effects of post-translational modifications of proteins in lysed cells, and some experiments require a high degree of purity in the activity of lysed proteins, making experimental manipulation difficult. Recent developments and optimizations in approaches using lysed cells are summarized and discussed.

Compared with traditional TPP, the mTSA method based on protein thermal stability differences demonstrates enhanced screening sensitivity with reduced cost and time consumption. The adoption of DIA instead of TMT-based quantification simplifies experimental procedures. Furthermore, the mTSA enables the calculation of ligand–target protein binding affinity, based on which the major target proteins bound by the ligand can be determined [44]. When thermal denaturation proves ineffective in inducing protein precipitation, the SPP method utilizing organic solvents for protein precipitation serves as a superior alternative, though some solvent-resistant proteins remain challenging [56]. The Solvent-PISA method further optimizes SPP by enabling the simultaneous analysis of various replicates and concentrations, enhancing operational simplicity and efficiency. The pHDPP method employs pH-gradient-induced protein denaturation, serving as an alternative strategy for studying proteins insensitive to both thermal and organic solvent denaturation [57]. The ipHSA method eliminates TMT labeling, thereby reducing costs [51]. The IPSSA method synergizes the ipHSA, iTSA, and iSSA approaches to achieve higher sensitivity and throughput in target screening. The PELSA method exhibits higher sensitivity than traditional proteolytic susceptibility-based target identification methods. It enables the detection of low-abundance proteins that are often masked under experimental conditions while providing information on the ligand’s binding region and local binding affinity data [58]. Lysine reactivity profiling preserves small-molecule activity by eliminating the need for ligand modification after probe–protein binding [23]. In summary, with these improvements, cell lysate-based methods exhibit significant advantages in target identification, establishing them as powerful tools for biological research and drug discovery.

Living cell experiments reproduce the cellular environment and molecular interactions, offering researchers more realistic and direct evidence of the physiological relevance and molecular interactions. The iTSA and TS-FITGE use protein thermal shifts to identify drug target proteins [59,116]. The former can search for ligand target proteins in various cell states; its simplified experimental design has the advantages of high sensitivity and reproducibility in high-throughput screening. TS-FITGE uses 2D gel electrophoresis to quickly distinguish the target protein [117]. I-PISA makes use of the principle that specific ions have the ability to change the solubility of proteins, which facilitates the recognition of target proteins of small-molecule drugs [63]. Tissue-TPP further expands the scope of the test sample, as it can directly test tissues or body fluids, is more in line with the metabolism of drugs in the body, and can also explain the off-target effect of drugs [61]. Compared with the addition of drug treatment to find target proteins in lysed cells, the use of intact cells as samples has greater guiding significance for the development of clinical drugs. Living cell experiments can be carried out in a natural intracellular environment, reproducing the structure and composition of cells, as well as the molecular interactions inside and outside cells, and simulating the real physiological state of cells in the body. Therefore, the results obtained are more physiologically relevant and biologically significant, providing a realistic and dynamic research platform for the search for drug targets or drug development.

High-throughput drug target identification approaches have been designed to optimize the experimental methodology and enhance the throughput of the target identification process. For example, MSIPP uses mechanical stress to precipitate proteins. Proteins are exposed to mechanical stress and denatured to induce aggregation on the surface of the particles. The combination of beads and proteins enables batch-processing samples, and this high-throughput processing facilitates the adjustment of experimental steps at any time to optimize the process [53]. Based on the principle that proteins in the same complex have similar thermal shift curves, TPCA uses the CETSA method to determine the dissolution curves of thousands of proteins and then classifies them based on the similarity between the thermal shift curves of these proteins, which can be used to monitor the dynamic changes in protein complexes in cells in a high-throughput manner and to detect the changes in protein interaction in different cell cycles and pathological conditions under different drug treatments [65]. DropScan mainly focuses on searching for drugs that affect phase separation related to cancer. The regulation of abnormal phase separation by small-molecule drugs is a potential strategy to treat related cancers [66,154]. High-throughput screening and the verification of PS-DBD fusion protein libraries using drugs can help identify drugs that can target phase separation, providing new insights for clinical drug development.

The label-free method mentioned in this study is mainly implemented with MS. Besides MS, biophysical techniques such as SPR, microscale thermophoresis (MST), isothermal titration calorimetry (ITC), and bio-layer interferometry (BLI) also enable the label-free characterization of molecular interactions via optical or thermodynamic signals [160,161,162]. In CETSAs, target engagement can be monitored using Western blot or ELISA to assess changes in protein solubility upon heating [135]. Similarly, protease resistance-based methods, including DARTS and LiP, rely on the protection of proteins against proteolysis upon ligand binding, whose outcomes can also be validated with Western blot [26,89]. NanoBRET-type target engagement assays detect energy transfer signals generated upon small-molecule–protein interactions through a principle similar to that of ELISA [163]. Non-mass spectrometric techniques enable the direct and quantitative characterization of protein–compound interactions based on thermodynamic, spectroscopic, or thermal stability-based readouts, with high specificity for validated target proteins. However, these approaches require preselected candidate proteins and do not support unbiased target identification. Furthermore, most assays rely on purified protein systems, are typically low-throughput, and can only analyze one protein–ligand interaction at a time. Moreover, certain methods are heavily dependent on antibody availability, yet high-quality antibodies are not accessible for all proteins. In contrast, mass spectrometry-based strategies require no prior knowledge of potential targets and enable the unbiased identification of ligand-binding proteins directly in complex biological samples, including intact cells, tissues, and cell lysates. These approaches are performed under near-physiological conditions and offer high throughput, allowing for the simultaneous profiling of thousands of proteins to capture a comprehensive landscape of candidate targets. However, such methods provide indirect evidence of protein–ligand interactions, as observed stability or solubility changes may arise from downstream regulatory effects or indirect drug-induced perturbations rather than direct binding. Accordingly, they cannot yield definitive conclusions alone and must be combined with non-mass spectrometry techniques to validate direct binding for individual target proteins. Collectively, these MS-independent approaches serve as core strategies to identify the targets of natural products. They can be combined with mass spectrometry-based methods for complementary validation, thereby advancing research from single-target validation to systematic multi-target and multi-pathway mechanistic studies.

The prediction of drug–target interactions has always been an important research area in drug discovery. With the development and advancement of proteomics, genomics, bioinformatics, and mass spectrometry, the ultimate goal is to continually improve the performance of such methods by integrating new technologies to reduce the cost and time of the assays and increase their throughput and accuracy. These new technologies will be used not only to identify natural product targets but also to elucidate the mechanisms of action and toxicity of natural products, and they will ultimately provide efficient ways to identify human disease markers and evaluate the therapeutic potential of natural products.

## Figures and Tables

**Table 1:** Newly identified targets of natural compounds determined by classical label-free methods.

Method	Natural Product	Newly Identified Target Protein	Mechanism of Action	Reference
Cellular context thermal shift assays (CETSAs)/ Thermal proteome profiling (TPP)	Gambogic acid (GBA)	Canopy FGF signaling regulator 3 (CNPY3)	Targeting CNPY3 lactic acid modification induces the pyroptosis pathway in prostate cancer cells, thereby inhibiting tumor proliferation and invasion.	[15]
	Deoxyshikonin	HSPA8-GEMIN5	Targeting the HSPA8-GEMIN5 complex interferes with mRNA splicing-translation coupling, destroys protein homeostasis, and induces apoptosis of colorectal cancer cells.	[17]
	Oridonin	Nucleolin	Targeted inhibition of ribosome biosynthesis induces tumor cell apoptosis.	[18]
	Quinine	Purine nucleoside phosphorylase of Plasmodium falciparum (PfPNP)	Inhibiting the catalytic activity of PfPNP and blocking the purine salvage synthesis pathway of Plasmodium leads to a lack of raw materials for nucleotide synthesis of Plasmodium and an inability to complete DNA replication and cell proliferation.	
Pulse proteolysis (PP)	Celastrol	Pyruvate kinase M2 (PKM2)	Targeting PKM2 allosteric sites to inhibit its kinase activity, block the glycolysis pathway of cancer cells, reduce the energy supply of cancer cells, and inhibit proliferation and invasion.	[16]
		Glyceraldehyde-3-phosphate dehydrogenase (GAPDH)	Targeting the GAPDH catalytic domain to inhibit the key steps of glycolysis, while inducing oxidative stress in cancer cells and triggering the mitochondrial apoptosis pathway.	
		AnnexinII (ANXA2)	Interfere with ANXA2-mediated cell adhesion and migration, inhibit the metastatic potential of colorectal cancer cells, and reduce tumor invasiveness.	
Drug affinity responsive target stability (DARTS)	Resveratrol	Eukaryotic initiation factor 4a (eIF4A), eukaryotic initiation factor 2 (eIF2)	Targeting eIF4A and eIF2 inhibits translation initiation and mediates longevity/anti-cancer effects.	[26]
	Laurifolioside	Clathrin heavy chain	Targeted inhibition of clathrin-mediated endocytosis and inflammatory signals regulation (such as NF-κB).	[29]
Stability of proteins from rates of oxidation (SPROX)	Staurosporine	Serine/threonine kinase family	Inhibit kinase phosphorylation activity, block cell cycle progression and proliferation signaling pathways, and exert anti-tumor activity.	[31]
Solvent-induced protein precipitation (SIP)	Geldanamycin (GA)	NADH dehydrogenase flavin protein 1 (NDUFV1)	Inhibit the catalytic activity of mitochondrial complex I and ultimately cause mitochondrial oxidative stress and damage.	[32]

**Table 2:** Summary of probe-free target identification methods.

Detection Method	Principle of Target Identification	Method	Target Identification Detection Method	Advantages	Limitations	Cell Line or Model Studied	Reference
Methods suitable for target identification in cell lysates	Differences in chemical denaturant–induced stability of protein targets	Solvent proteome profiling (SPP)	LC-MS/MS	Suitable alternative for cases where binding to the target protein does not respond to thermal denaturation.	Not suitable for cell-based methods, as solvent acetic acid (AEA) cannot enter living cells and thus cannot induce protein precipitation; some proteins are not sensitive to treatment with organic solvents.	HCT116 cells	[56]
		Solvent proteome integral solubility alteration (Solvent-PISA)	LC-MS/MS	It allows for the simultaneous analysis of multiple replicates, multiple concentrations, or multiple compounds.		HCT116 cells	
	Differences in pH-induced stability of protein targets	pH-dependent protein precipitation (pHDPP)	LC-MS/MS	It addresses the disadvantage that some proteins do not respond to thermal denaturation.	Higher cost of TMT labeling; long processing time.	HeLa and 293T cells	[57]
		Iso-pH shift assay (ipHSA)	DIA-MS	More efficient operation without TMT labeling; high throughput and reproducibility of DIA-MS analytical methods.	Only one compound can be verified in a single experiment.	K562 cells	[51]
		Integrated protein solubility shift assay (IPSSA)	DIA-MS	Higher sensitivity and improved throughput compared with ipHSA, iTSA, and iSSA methods.	The experimental procedure is relatively complex.	K562 cells	
	Differences in the thermal stability of protein targets	Matrix thermal shift assay (mTSA)	DIA-MS	Higher sensitivity than TPP; binding affinity can be calculated simultaneously from the dose response spectrum of individual targets.	Some proteins do not respond to thermal denaturation.	K562 and HepG2 cells	[44]
	Differences in proteolytic stability induced by ligand binding	Peptide-centric local stability assay (PELSA)	DIA-MS	Higher sensitivity to capture weakly altered low-abundance proteins; it provides information on ligand-binding regions.	There is a bias in identifying membrane proteins; large amounts of trypsin must be used.	HeLa, K562, BT474 and Jurkat cells	[58]
	Differences in amino acid reactivity of ligand-bound target proteins	Lysine reactivity profiling	DIA-MS	It combines the advantages of high sensitivity in finding the target and maintaining the activity of small-molecule compounds without the need for probe modification.	It still involves the use of probes and is more costly and harder to implement.	K562 cells	[23]
Methods suitable for target identification in living cells	Differences in the thermal stability of protein targets	Isothermal shift assay (iTSA)	nanoLC-MS/MS	Improved throughput; it requires a lower sample dose than TPP.	Higher cost of TMT labeling; longer experimental cycles.	K562 cells or the mouse cortex	[59]
		Thermal stability shift-based fluorescence difference in two-dimensional gel electrophoresis (TS-FITGE)	2D gel electrophoresis	Rapid exclusion of non-target proteins; membrane-anchored proteins can be identified.	Unable to identify low-abundance proteins; some proteins do not respond to thermal denaturation.	HEK293T cells	[60]
		Tissue-thermal proteome profiling (Tissue-TPP)	LC–MS/MS	It can be applied directly in tissues for detection and can account for off-target effects of drugs.	Tissue samples are complex and heterogeneous; some proteins do not respond to thermal denaturation.	Various tissue types	[61]
		Proteome integral solubility alteration (PISA)	LC-MS/MS	Independent of a single-temperature-point selection, and with fewer false negatives.	Only the solubility change multiple can be obtained, and the thermal stability change cannot be accurately quantified; hard to distinguish between direct binding and indirect stability.	A549 cells and A498 cells	[62]
	Differences in ion-induced stability of protein targets	Ion-based proteome-integrated solubility alteration (IPISA)	LC-MS/MS	Samples can be mixed together, and throughput is improved.	Sensitivity is reduced due to sample merging.	K562 cells	[63]
High-throughput schemes for finding target compounds	Differences in physical factors-induced stability of protein targets	Mechanical stress-induced protein precipitation (MSIPP)	LC-MS/MS	It identifies membrane-anchored proteins; precipitated proteins are physically bound to particles for more precise results; high-throughput operation can be performed using an orifice plate.	It cannot be used with living cells; some proteins are not easily captured by particles or require greater mechanical stress to be precipitated.	Hela and K562 cells	[53,64]
	Differences in thermal stability of protein targets	Thermal proximity co-aggregation (TPCA)	LC–MS/MS	Proteins in aggregation complexes can be studied.	Low-abundance proteins are not easily captured; they require explicitly interacting proteins as positive controls; a larger sample size of protein is needed; it relies on a high-quality quantitative mass spectrometry platform.	K562 cells	[65]
	-	DropScan	Phenotypic Observations	Rapid screening for relevant acting drugs based on cancer-associated PS-DBD fusion proteins.	PS-DBD library construction is cumbersome and does not enable the discovery of new cancer-associated phase-separated proteins.	PS-DBD fusion protein library	[66]

## Data Availability

No new data were created in this study, and all information is already included in the article.
